# Supplementary figures and images for: Development of AlissAID system targeting GFP or mCherry fusion protein
Source: PLoS Genet. 2023 Jun 14;19(6):e1010731. doi: 10.1371/journal.pgen.1010731 (PMC10266622; doi:10.1371/journal.pgen.1010731)

A

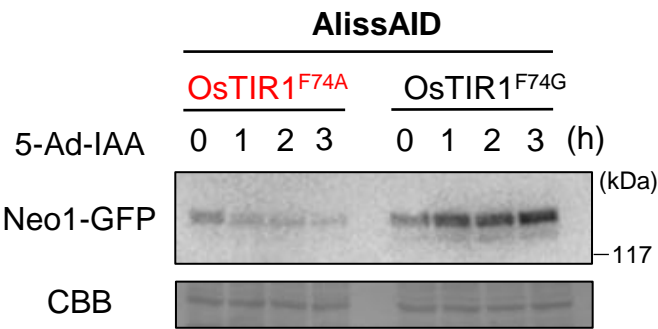

B

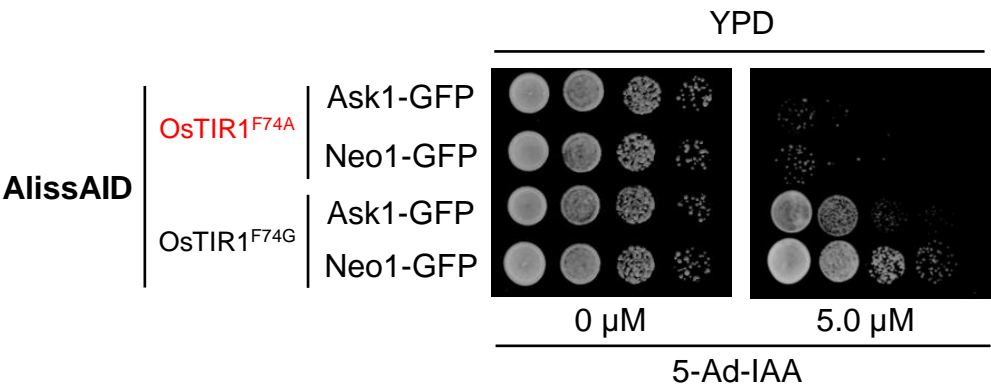

Supplement: S1 Fig — (A) Immunoblots of AlissAID strains that had OsTIR1 (OsTIR1 F74A or OsTIR1 F74G), showing the 5-Ad-IAA-triggered degradation of a target protein (Neo1-GFP). CBB-stained proteins were used as a loading control. (B) Serial dilution spotting assay of AlissAID strains on YPD medium. Each strain that had an essential protein (Ask1-GFP or Neo1-GFP) as a target protein was grown with or without 5-Ad-IAA at 30°C for 24 h. (PDF) [file pgen.1010731.s001.pdf]

**A**

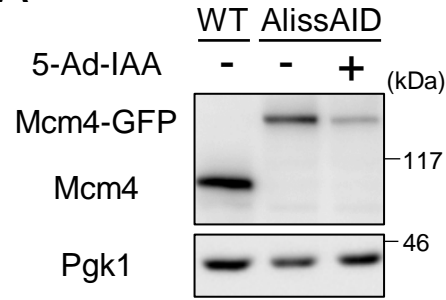

**B**

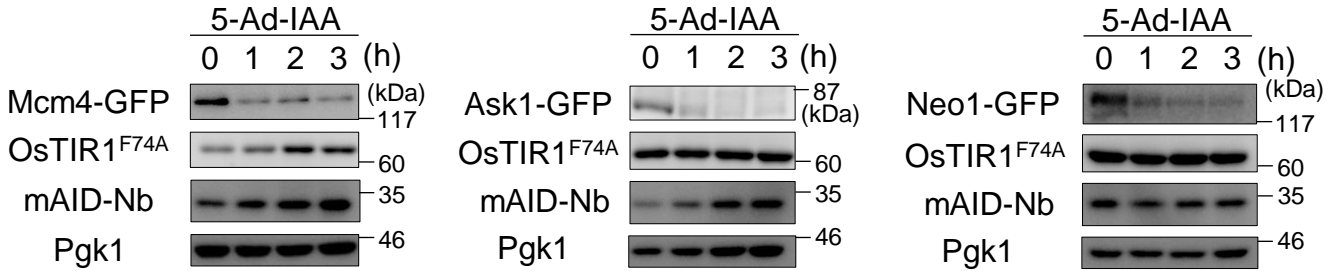

**C**

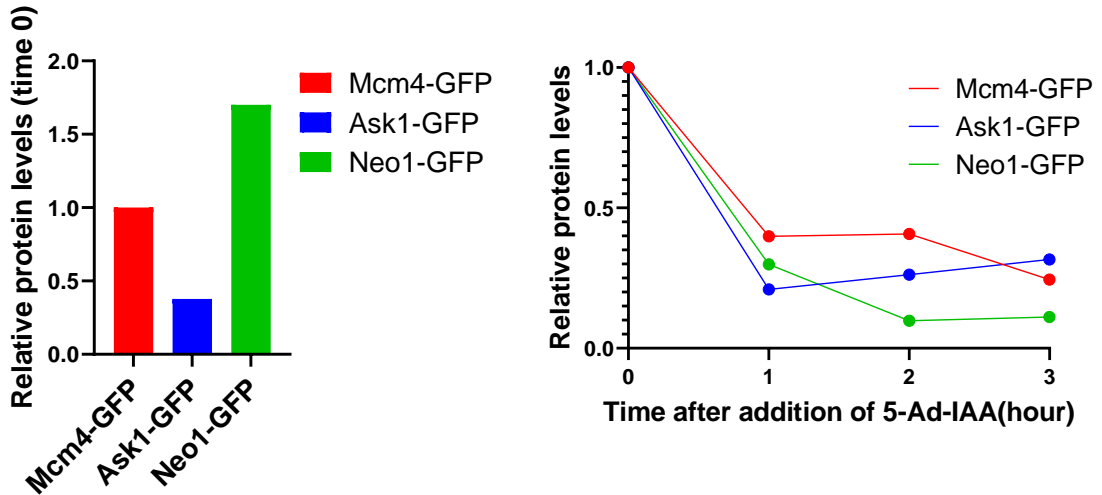

**D**

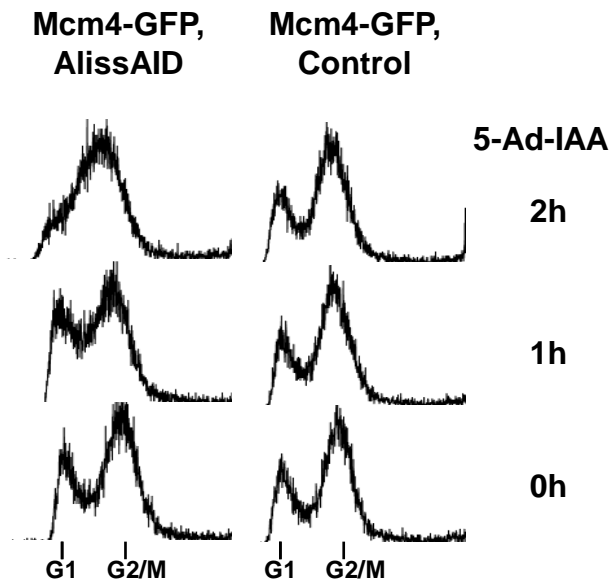

**E**

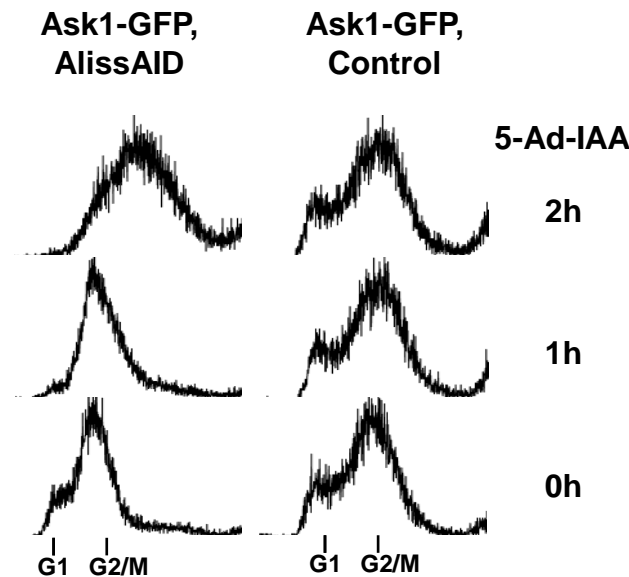

Supplement: S2 Fig — (A) Immunoblot of WT and Mcm4-AlissAID strain by the anti-Mcm4 antibody. Cells were treated with 5μM 5-Ad-IAA for 1h. Pgk1 was used as a loading control. (B) Immunoblots of AlissAID strains toward Mcm4, Ask1 and Neo1. Cells were treated with 5 μM 5-Ad-IAA for 0, 1, 2, and 3 h. (C) Expression levels of Mcm4, Ask1 and Neo1 proteins in the absence of 5-Ad-IAA. Signal intensities of Mcm4-GFP, Ask1-GFP and Neo1-GFP bands were normalized by using the loading control Pgk1. (D) Degradation profiles of Mcm4, Ask1 and Neo1 in AlissAID system. Signal intensities of Mcm4-GFP, Ask1-GFP and Neo1- GFP bands were normalized by using the loading control Pgk1. Relative levels of these proteins at time zero are shown. (E) Cell cycle profiles of Mcm4- and Ask1-AlissAID strains. Cells were treated with 5 μM 5-Ad-IAA for 0, 1 and 2 h. (PDF) [file pgen.1010731.s002.pdf]

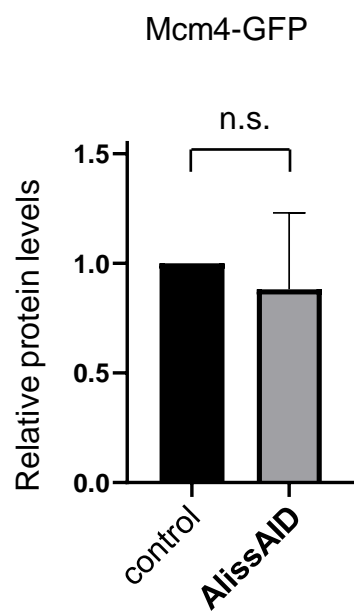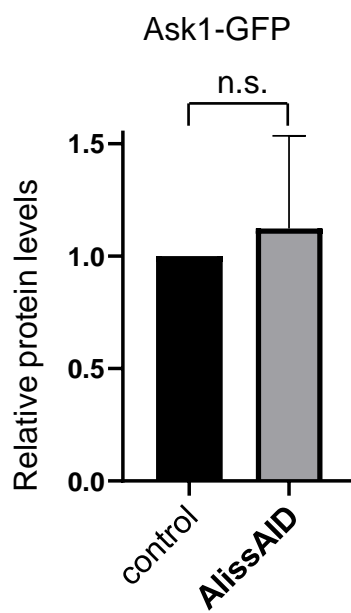

Supplement: S3 Fig — Comparison of the levels of a target protein (Mcm4-GFP or ASK-GFP) in AlissAID strain with those in the control strain that had the respective target protein, but neither OsTIR1 nor mAID-Nb. After normalizing signal intensities on the immunoblot with the loading control Pgk1, target protein levels were statistically analyzed. Means ± SD (n = 3 biological replicates); n.s., nonsignificant; unpaired Student’s t-test. (PDF) [file pgen.1010731.s003.pdf]

A

## AlissAID

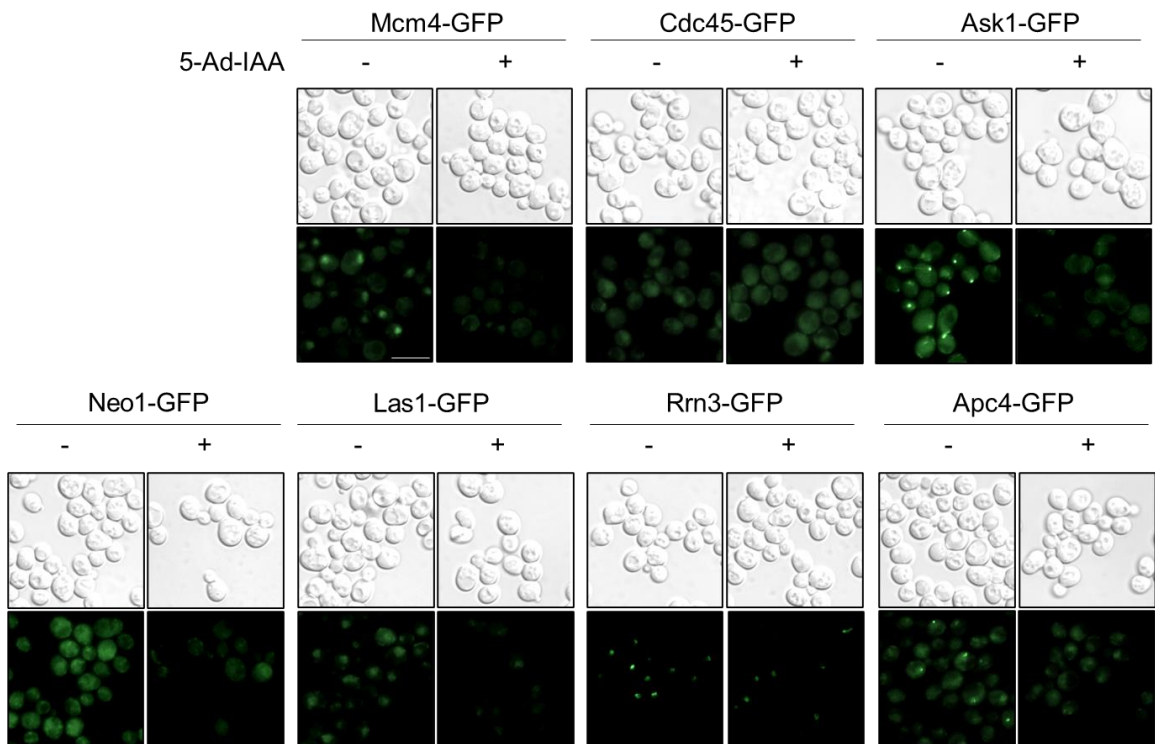

B

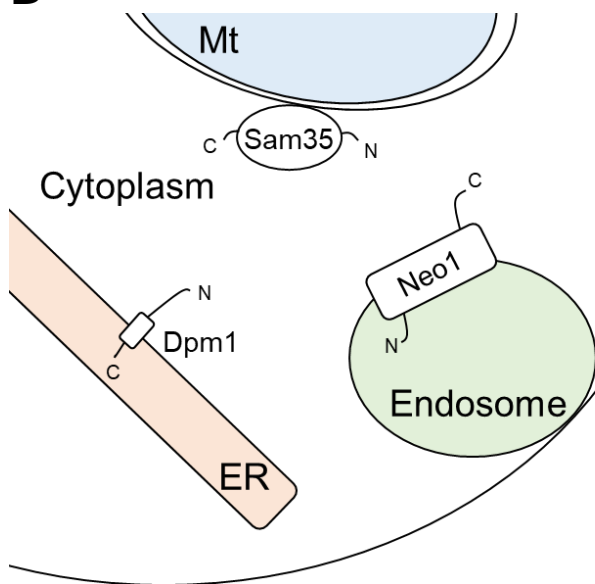

C

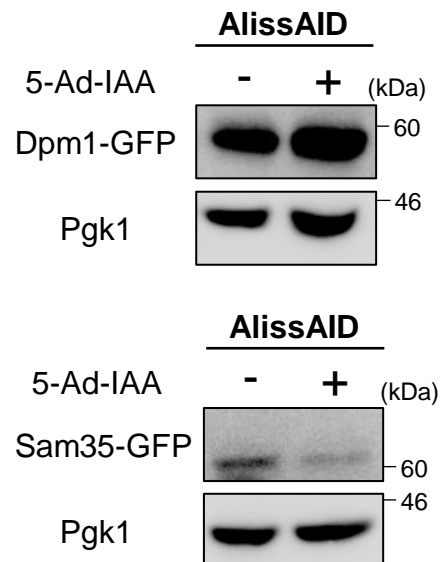

D

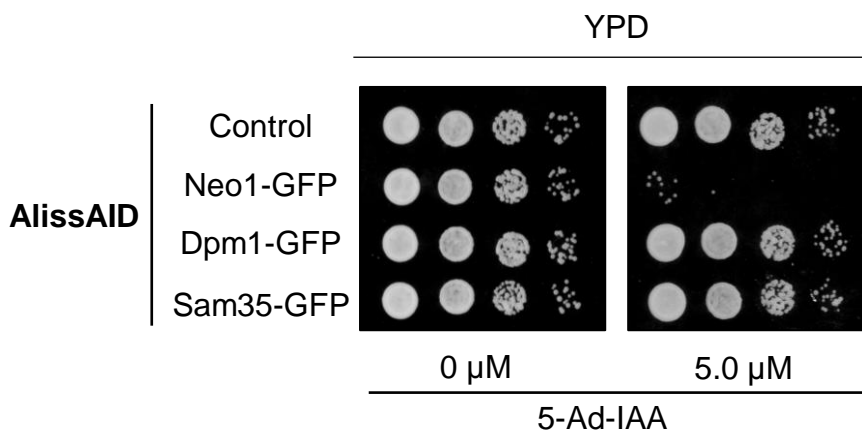

Supplement: S4 Fig — (A) Fluorescence microscopy observations of AlissAID strains toward Mcm4, Cdc45, Ask1, Neo1, Las1, Rrn3 and Apc4. These AlissAID strains were generated from GFP clone collection. Cells were treated with or without 5.0 μM 5-Ad-IAA for 3 h. Scale bar, 5 μm. (B) Schematic diagram of subcellular localization of Neo1, Dpm1 and Sam35. (C) Immunoblots of Dpm1- and Sam35-AlissAID strain. Cells were treated with 5 μM 5-Ad-IAA for 1h. Pgk1 was used as a loading control. (D) Serial dilution spotting of the control, Neo1-, Dpm1- and Sam35-AlissAID strains on YPD medium with or without 5 μM 5-Ad-IAA. Cells were grown for 24 h at 30°C. (PDF) [file pgen.1010731.s004.pdf]

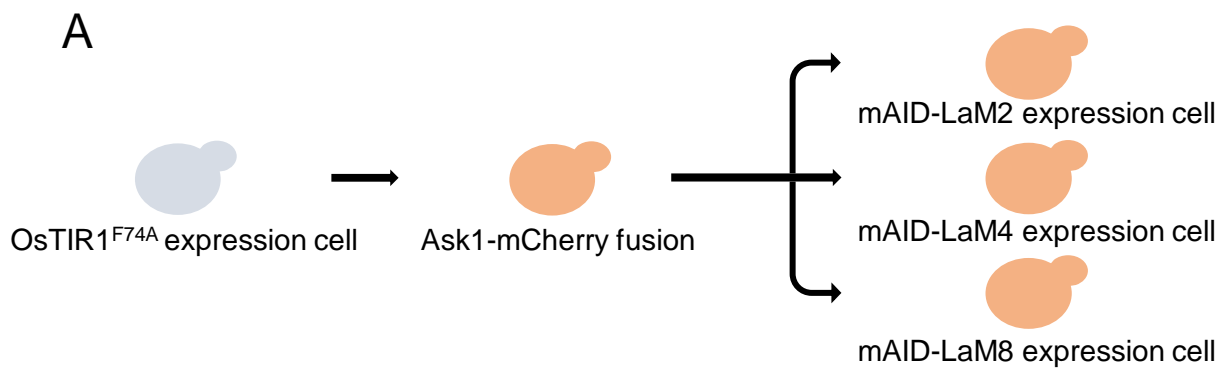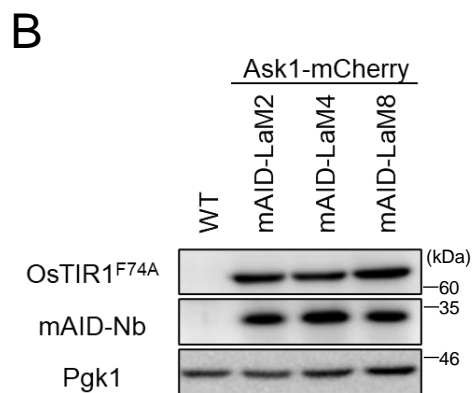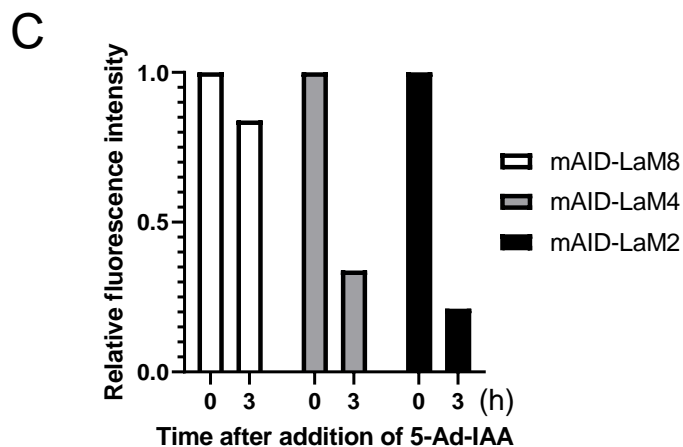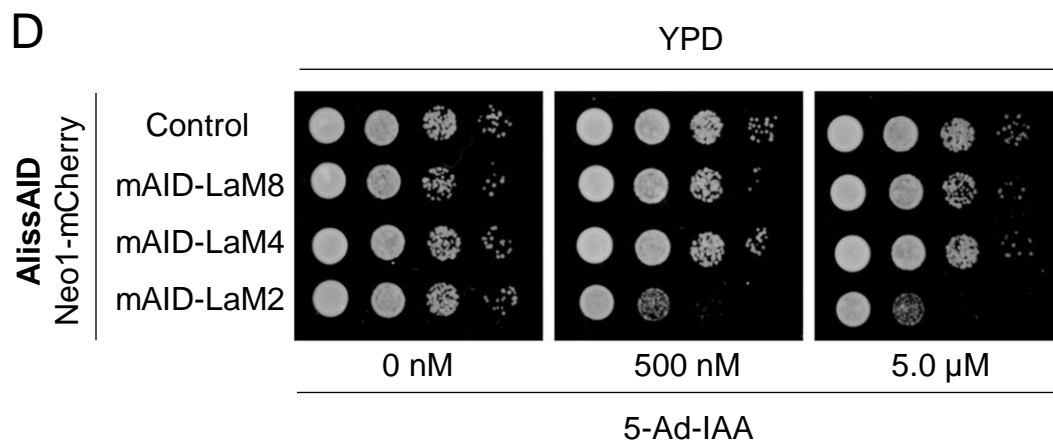

Supplement: S5 Fig — (A) Schematic illustration for generation of Ask1-AlissAID stains, which express mAID-LaMs (anti-mCherry nanobody). All LaM-expressing cell lines were established from mCherry tagged cell lines to the same OsTIR1F74A-expressing line. (B) Immunoblots of three AlissAID strains, each of which had mAID-Nb (LaM2, LaM4, or LaM8) and a target protein (Ask1-mCherry). Pgk1 was used as a loading control. (C) Fluorescence intensities of Ask1-mCherry in three independent AliceAID strains that were treated with 5-Ad-IAA for 0 and 3 h. Fluorescence intensities were acquired from the images in Fig 6B by Image and were normalized with the number of cells in the images. (D) Serial dilution spotting assay of AliceAID strains on YPD medium. Each strain that had mAID-Nb (LaM2, LaM4, or LaM8) and an mCherry-tagged essential protein (Neo1-mCherry-Flag) as a target protein was grown with 5-Ad-IAA at 30°C for 24 h. (PDF) [file pgen.1010731.s005.pdf]
