# Supplementary material for: Development of AlissAID system targeting GFP or mCherry fusion protein
Source: PLoS Genet. 2023 Jun 14;19(6):e1010731. doi: 10.1371/journal.pgen.1010731 (PMC10266622; doi:10.1371/journal.pgen.1010731)
Supplement: S1 Table — (PDF) [file pgen.1010731.s006.pdf]

Name	Genotype	Source
W303-1a	*MAT*a *leu2-3,112 trp1-1 can1-100 ura3-1 ade2-1 his3-11,15*	
YYS100	W303-1a, *ASK1-GFP::HIS3MX6*	This study
YYS101	W303-1a, *NEO1-GFP::HIS3MX6*	This study
YYS105	W303-1a, *ADH1p-OsTIR1(F74A)::URA3,ADH1p-mAID(KR)-VHHGFP4(KR)::LEU2*	This study
YYS107	W303-1a, *ADH1p-OsTIR1(F74A)::URA3,ADH1p-mAID(KR)-VHHGFP4(KR)::LEU2, ASK1-GFP::HIS3MX6*	This study
YYS108	W303-1a, *ADH1p-OsTIR1(F74A)::URA3,ADH1p-mAID(KR)-VHHGFP4(KR)::LEU2, NEO1-GFP::HIS3MX6*	This study
YYS109	W303-1a, *ADH1p-OsTIR1(F74G)::URA3,ADH1p-mAID(KR)-VHHGFP4(KR)::LEU2, ASK1-GFP::HIS3MX6*	This study
YYS110	W303-1a, *ADH1p-OsTIR1(F74G)::URA3,ADH1p-mAID(KR)-VHHGFP4(KR)::LEU2, NEO1-GFP::HIS3MX6*	This study
YYS115	W303-1a, *ADH1p-OsTIR1(F74A)::URA3,ADH1p-mAID(KR)-VHHGFP4(KR)::LEU2, MCM4-GFP::HIS3MX6*	This study
YYS117	W303-1a, *MCM4-GFP::HIS3MX6*	This study
YYS149	W303-1a, *NEO1-mAID-GFP::His3MX6*	This study
YYS156	W303-1a, *ADH1p-OsTIR1(F74A)::URA3,ASK1-mCherry::HIS3MX6*	This study
YYS158	W303-1a, *ADH1p-OsTIR1(F74A)::URA3,ADH1p-mAID(KR)-Lam2(KR)::LEU2,ASK1-mCherry::HIS3MX6*	This study
YYS159	W303-1a, *ADH1p-OsTIR1(F74A)::URA3,ADH1p-mAID(KR)-Lam4(KR)::LEU2,ASK1-mCherry::HIS3MX6*	This study
YYS160	W303-1a, *ADH1p-OsTIR1(F74A)::URA3,ADH1p-mAID(KR)-Lam8(KR)::LEU2,ASK1-mCherry::HIS3MX6*	This study
YYS184	W303-1a, *ADH1p-OsTIR1(F74A)::URA3,ADH1p-mAID(KR)-Lam2(KR)::LEU2,NEO1-mCherry-Flag::HIS3MX6*	This study
YYS185	W303-1a, *ADH1p-OsTIR1(F74A)::URA3,ADH1p-mAID(KR)-Lam4(KR)::LEU2,NEO1-mCherry-Flag::HIS3MX6*	This study
YYS186	W303-1a, *ADH1p-OsTIR1(F74A)::URA3,ADH1p-mAID(KR)-Lam8(KR)::LEU2,NEO1-mCherry-Flag::HIS3MX6*	This study
YYS191	W303-1a, *ADH1p-OsTIR1(F74A)::URA3,ADH1p-mAID(KR)-VHHGFP4(KR)::LEU2, DPM1-GFP::HIS3MX6*	This study
YYS196	W303-1a, *ADH1p-OsTIR1(F74A)::URA3,ADH1p-mAID(KR)-VHHGFP4(KR)::LEU2, SAM35-GFP::HIS3MX6*	This study
BY4741	*MATa his3Δ1, leu2Δ, ura3Δ, met15Δ*	
YYS53	BY4741, *CDC45-GFP::HIS3MX6*	GFP Clone Collection(Thermo Fisher Scientific)
YYS54	BY4741, *MCM4-GFP::HIS3MX6*	GFP Clone Collection(Thermo Fisher Scientific)
YYS56	BY4741, *APC4-GFP::HIS3MX6*	This study
YYS67	BY4741, *LAS1-GFP::HIS3MX6*	GFP Clone Collection(Thermo Fisher Scientific)
YYS78	BY4741, *ASK1-GFP::HIS3MX6*	This study
YYS80	BY4741, *NEO1-GFP::HIS3MX6*	This study
4602	BY4741, *RRN3-GFP::HIS3MX6*	Our Laboratory
YYS177	BY4741, *MCM4-GFP::HIS3MX6,ADH1p-OsTIR1(WT)-T2A-mAID-VHHGFP4::URA3*	This study
YYS178	BY4741, *MCM4-GFP::HIS3MX6,ADH1p-OsTIR1(F74A)-T2A-mAID-VHHGFP4::URA3*	This study
YYS179	BY4741, *MCM4-GFP::HIS3MX6,ADH1p-OsTIR1(F74S)-T2A-mAID-VHHGFP4::URA3*	This study
YYS180	BY4741, *MCM4-GFP::HIS3MX6,ADH1p-OsTIR1(F74C)-T2A-mAID-VHHGFP4::URA3*	This study
