# Supplementary material for: Development of AlissAID system targeting GFP or mCherry fusion protein
Source: PLoS Genet. 2023 Jun 14;19(6):e1010731. doi: 10.1371/journal.pgen.1010731 (PMC10266622; doi:10.1371/journal.pgen.1010731)
Supplement: Attachment — Submitted filename: Response to Reviewers (PGENETICS-D-22-01432).docx [file pgen.1010731.s008.docx]

**Reviewer #1:**

**Summary:**
In this study, Ogawa and Nishimura et al. developed an affinity linker-based super sensitive auxin-inducible degron (AlissAID) by using a single domain antibody (nanobody) in a budding yeast. This system can degrade the desired target proteins by administering a synthetic auxin, namely, 5-adamantyl-IAA (5-Ad-IAA), at nanomolar concentrations. To prove the usefulness of this system, the authors utilized it for the degradation of several essential proteins. They also developed the “AlissAID plasmid,” a single plasmid that encodes both OS TIR1 (F74A) and mAID (KR)-nanobody (KR) and may be used to systematically degrade GFP-tagged proteins. Finally, the authors demonstrated that this system could be applied to eliminate mCherry-fused proteins by using the nanobody that specifically recognizes mCherry. Overall, the AlissAID system would be a highly efficient tool for the genetic analysis of budding yeasts and other higher eukaryotes.

**Major comments:**
The authors assessed the degradation of GFP-fused target proteins by western blotting with the GFP antibody and confirmed it by analyzing cell growth and cell cycle progression. However, these analyses are indirect. It is formally possible that only the GFP moiety was degraded, and a portion of target proteins likely remained intact. Therefore, the authors should demonstrate the extent of target protein degradation by using an endogenous antibody or other epitope tags at the N-terminus (e.g., HA, myc, or FLAG). This aspect is important because this study is the first to characterize the novel protein knockdown system.

**Authors’ Responses**

We appreciate the comment. As suggested, we did additional experiments with specific antibodies against the target protein Mcm4. We provided the result showing the degradation of the portion of the target protein (Fig. S2A) and the explanation in the text (Lines 146-147).

**Minor comments:**
1. In Fig. 2A, the mAID-Nb level increased after 5-Ad-IAA was administered in Mcm4-GFP cells and Ask1-GFP cells but not in Neo1-GFP cells. Please provide an explanation for these results.

**Authors’ Responses**

In our experiments, the mAID-Nb levels were not always increased. To avoid misleading of the readers, we provided the new immunoblots in new Fig. 3A and the previous immunoblots (Fig 2A) in Fig. S2B.

2. In Fig. 2C, the cells expressing OSTIR1 (F74A) and mAID-Nb seem to grow better than the control cells do. Is there any reason?

**Authors’ Responses**

It was reported that control strains (auxotrophic strains) show weak growth defects in YPD medium (DOI 10.1007/s10482-010-9530-5). According to the report, control strains might less efficiently utilize nutrients than AlissAID strains that had two selection markers (*URA3* and *LEU2* genes).

3. In Fig. 3C, “hour” may be removed from “5-Ad-(IAA)(hour).”

**Authors’ Responses**

Done.

4. In Fig. S1, the authors need to show the FACS data of 5-AD-IAA-treated cells that do not express OSTIR1F74A and mAID-Nb.

**Authors’ Responses**

As suggested, we did additional FACS experiments of 5-Ad-IAA-treated cells that expressed neither OsTIR1F74A nor mAID-Nb. We then provided the results in Supplementary Fig. 2D and 2E and the explanation in the text (Lines 157-161).

In addition, the profile of the DNA content of Mcm4-GFP cells at 0 h likely differs from that of Ask1-GFP cells. Does the attachment of GFP to Ask1 affect the cell cycle?

**Authors’ Responses**

　　 Yes, the attachment of GFP to Ask1 affects the cell cycle. Ask1-GFP cells are arrested at G2/M phase.

5. The authors tested this system only at 30 °C. Can this system be used at other (low and high) temperatures?

**Authors’ Responses**

We additionally tested it at 20 ˚C, 24 ˚C, and 37 ˚C and then provided the results in Fig. 3D and the explanation in the text (Line 155-157). The results clearly show that AlissAID system is useful in a wide range of temperatures.

6. The methods of statistical analyses should be presented more clearly in both the Methods section and Figure legends. For example, in Fig. 3 and 5, do error bars stand for standard deviation or standard error? How were the protein levels normalized? How was the p-value calculated? What was the statistical method used?

**Authors’ Responses**

As suggested, we added the new subsection “Statistical analysis” in Materials and Methods and described the detailed method. We also added the explanation of statistical analysis of the data in the legends of Fig. 2 (previous Fig. 5) and Fig.4 (previous Fig.3).

7. The molecular weight (MW) is shown in some blots only. The authors should show the MW markers in all blots.

**Authors’ Responses**

Done.

**Reviewer #2:** 
**Introduction**
Provided a succinct yet thorough background on past AID advances and limitations, and the logical next step of merging the ssAID and AID-tagged nanobodies approaches into a single approach.

Line 73:” that OsTIR1F74A showed a mild sensitivity to IAA” could the authors clarify precisely what type of sensitivity they are referring to here? It was a little unclear since the previous sentence referred to basal degradation but this is clearly talking about in the presence of IAA.

**Authors’ Responses**

To clarify the statement, we improved the part as follows (see also Lines 72-77):

＜Lines 72-77＞

Some improved AID systems (including ssAID and AID2) showing limited basal degradation have been developed in mammalian cell lines [11, 12, 16, 17] and fission yeast [13]. However, IAA induced a weak interaction between OsTIR1^F74A^ and AID-tag in the yeast two hybrid assay [11].Therefor, it is possible that basal degradation is an issue to be solved in AID system of budding yeast.

**Results**
Fig 1: is a useful visual to understand the differences between the various AID systems referred to in this work, and could actually be cited when they come up in the Introduction too. Please include in 1C a label for the nanobody component, and consider shortening “AID-tag (IAA17)” to “mAID” for simplicity?

**Authors’ Responses**

Done.

Fig 2: in 2C the (-) sign was not obvious to me at first, it could be made a bit bigger or bolder and then rather than the “OsTIR1F74A, mAID-nb” label that takes up two lines, simplify to “AlissAID” so that it will fit on a single line. I suggest that in this and all other figures, the authors consider using the simplified label “AlissAID” when possible to decrease the amount of text on the figures.

**Authors’ Responses**

Done.

In S1 referred to at line 132, it actually looks like very little effect at 1 hr for Mcm4, but at later time points both Mcm4 and Ask1 actually show G2/M arrest?

**Authors’ Responses**

To clarify the point, we did additional FACS experiments of control cells that expressed neither OsTIR1F74A nor mAID-Nb. The results showed that Mcm4-GFP and Ask1-GFP cells started to be arrested at S phase and at G2/M phase 1 h after adding 5-Ad-IAA, respectively. We provided the results in Supplementary Fig. 2D and 2E, and revised the text (Lines 157-161).

＜Lines 157-161＞

Mcm4 and Ask1 are essential for the progression of DNA replication in S phase and for chromosomal segregation in G2/M phases, respectively [29, 30]. FACS analysis showed that Mcm4-GFP cells and Ask1-GFP cells started to be arrested at S phase and at G2/M phase 1 h after adding 5-Ad-IAA, respectively (S2D and S2E Figs).

Fig 3: Lines 143-145 This rationale feels like it comes out of nowhere describing a key piece of data before showing that data…maybe the authors could reframe it by rearranging to lead with the sentence “There are questions about..”, followed by “To approach this…” and then “We coincidentally found…”

**Authors’ Responses**

As suggested, we improved the part (Lines 166-172).

＜Lines 166-172＞

There are questions about the extent to which basal degradation occurs in AlissAID system of budding yeast. To approach this, we evaluated basal degradation in the ssAID and AlissAID systems of budding yeast. We generated two strains: (i) an Neo1-ssAID strain based on OsTIR1^F74A^-expressing cell, in which C terminus of Neo1 was tagged by mAID-GFP and (ii) an Neo1-AlissAID strain based on OsTIR1^F74A^-and-mAID-Nb-expressing cell, in which C terminus of Neo1 was tagged by GFP. We observed a substantial reduction in Neo1 protein levels even in the absence of 5-Ad-IAA in the ssAID system (Fig 4A).

Again, the authors could simplify the labels to AlissAid and ssAID.

**Authors’ Responses**

Done.

Line 152 - before this sentence, flow could be improved by explicitly stating rationale here, for example something like “To confirm that this is due to basal degradation due to presence of OsTIR1, we also built strains lacking receptors…”

**Authors’ Responses**

As suggested, we improved the part (Lines 173-179).

＜Lines 173-179＞

This reduction would be due to the basal degradation. To confirm it, we further generated the control strain lacking OsTIR1^F74A^. Neo1 protein was degraded with 5-Ad-IAA treatment in AlissAID and ssAID strains, but not in the control strains (Fig 4B and 4C). Importantly, even in the absence of 5-Ad-IAA, the expression levels of Neo1-mAID-GFP were reduced in ssAID strain (Fig 4C). On the other hand, the expression levels of Neo1-GFP in the AlissAID strain did not change in the absence of 5-Ad-IAA (Fig 4B). We obtained similar results with Mcm4 and Ask1 in AlissAID strain (S3 Fig).

In 3C, there are two Neo1-GFP bands in each lane in each set of strains - which one is being quantified? The Neo1-GFP bands are hard to resolve in the Neo1-GFP strains, and expression does not match above in A at 0 inducer (i.e. expression is very strong in the blots in A compared to the Neo1-mAID-GFP, but the reverse seems to be true in the blots in C?). Did the blots not shown from the other two independent experiments show this or a different trend? Is there some degree of variability in expression level?

**Authors’ Responses**

In cases where multiple target bands appeared, all of the positive signals were quantified. We provided the information in “Statistical analysis” of Materials and Methods.

The Neo1-GFP and Neo1-mAID-GFP bands were detected on different blots, which causes a difficulty in comparing their signal intensities. To avoid misleading, we provided each immunoblot data in different panels together with the respective quantitative data: Neo1-GFP in Fig. 4B and Neo1-mAID-GFP in Fig. 4C.

Fig 4: At Line 558, the legend could use a bit more description of the 4A cartoon: what is depicted in top vs bottom (i.e. multistep vs single step process).

**Authors’ Responses**

As suggested, we improved the cartoon in Fig. 5A (previous Fig. 4A) and the legend.

Fig 5: Maybe this Figure and section (Lines 184-197) would make more sense right after Fig 1? Since most of the remainder of the paper uses the F74A mutation, this provides rationale for that choice throughout.

**Authors’ Responses**

According to the suggestion, we moved “Fig. 5” right after Fig. 1.

Fig 6: At Line 207, maybe briefly mention the difference between LaMs 2,4,and 8 to provide some rationale for why all three were tested?

**Authors’ Responses**

As suggested, we briefly mentioned the difference between LaMs 2,4, and 8 in the text (Lines 220-223).

＜Lines 220-223＞

Similar to GFP, the red fluorescent protein mCherry is commonly used in budding yeast and some anti-mCherry nanobodies (named LaMs, [31]) are available. We selected three LaMs (LaM2, LaM4 and LaM8) that have different properties in binding sites and binding forces.

In S4C, please clarify in the legend (Lines 524-526) that this is the quantification of the data from Fig 6b? Also, it looks like labels might be mixed up as Lam2 shows no degradation but microscopy image in 6b shows strong degradation. In S4D, the Lam2 data does not match data in C?

**Authors’ Responses**

We improved the legend of the Fig. S5C (previous S4C) to show that the quantification data was originated from the data of Fig. 6B (Lines 559-562).

We corrected the mistake of the labels of Fig. S5D (previous S4D).

＜Lines 559-562＞

(C) Fluorescence intensities of Ask1-mCherry in three independent AliceAID strains that were treated with 5-Ad-IAA for 0 and 3 h. Fluorescence intensities were acquired from the images in Fig. 6B by ImageJ and were normalized with the number of cells in the images.

**Discussion**
With the exception of the comment below, I found the Discussion to be straightforward and especially appreciated the exciting possibilities for future applications laid in the last paragraph. I found the second paragraph (Lines 230-245) a bit challenging to follow and had to re-read several times to try to make sense of what was being said. Some of the sentences are fragmentary, and I think this paragraph would benefit from some language editing. At 234, I think this is citing a different paper?

**Authors’ Responses**

According to the suggestion, we completely revised the second paragraph in the Discussion (Lines 246-250).

＜Lines 246-250＞

In this study, we found that the AlissAID system shows milder basal degradation than the ssAID system. It might be due to their different methods for the interaction between OsTIR1^F74A^ and the target protein: AlissAID system uses an indirect interaction via mAID-Nb, while ssAID system uses a direct interaction. Therefore, AlissAID system would be a good inducible degron system for the proteins that will be destabilized by AID tagging.

Methods
Very detailed and straightforward. Will the plasmids be made available through a repository such as Addgene?

**Authors’ Responses**

Yes, these plasmids are available from Addgene (https://www.addgene.org/). We added “Plasmid deposition” in Materials and Methods (Lines 369-373).

＜Lines 369-373＞

**Plasmid deposition**

These plasmids are available from Addgene. pRS305-mAID-Nb (VHHGFP4): #198412, pRS305-mAID-LaM2: #198413, pRS305-mAID-LaM4: #198414, pRS305-mAID-LaM8: #198415, pRS306-OsTIR1^F74A^: #198416, pRS316-OsTIR1^F74A^-T2A-mAID-Nb (VHHGFP4): #198417 (Line 370-374)

**Miscellaneous**
The paper is generally well-written but I do recommend some language editing for minor grammar and sentence structure revisions.

**Authors’ Responses**

According to the suggestion, we carefully revised the text and the figure legends.

**Reviewer #3:**

**Specific critique:**
1. Yeasts are divided every 60-90 minutes; therefore, 1h degradation could be too slow compared to the speed of cellular processes in the yeast cell. It will be essential to test a more detailed (10, 20, 30, 45 min) time course of degradation presented in Figure 1.

**Authors’ Responses**

We did additional experiments to test more detailed (15, 30, 45 min) time course. We provided the results in Fig. 2A. We also revised the legend (Lines 561-563) and the text (Lines 144-146).

2. To make the AlissAID system more universal, it will be important to compare the time course of degradation for both high- and low-expressed yeast proteins.

**Authors’ Responses**

We compared the degradation profiles of low-expressed protein (Ask1), middle-expressed protein (Mcm4), and high-expressed protein (Neo1). These degradation efficiencies were almost similar levels. We provided the result in Fig.S2C. we also revised the legend (Lines 532-534) and the text (Lines 147-150).

＜Lines 147-150＞

Next, we compared the degradation efficiency of target proteins with different expression levels. The degradation efficiencies of Mcm4 (middle expression), Ask1 (low expression) and Neo1 (high expression) are almost similar in this AlissAID system (S2B and S2C Figs).

It will also be essential to compare the degradation of FP-tagged protein localized in different compartments (cytosol, nucleus, peripheral and integral components of ER, Golgi, mitochondria, peroxisomes, vacuoles and plasma membrane).To compare the time course of degradation for high- and low-expressed yeast proteins, we compared the expression levels of Neo1-GFP, Mcm4-GFP and Ask1-GFP by using anti-GFP antibody.

**Authors’ Responses**

We did additional experiments of FP-tagged proteins localized in different compartments and provided the results in Fig. S4B, S4C, and S4D. We also provided the legends (Lines 548-553) and the explanation in the text (Lines 206-216).

＜Lines 206-216＞

We next examined GFP-tagged proteins localized in different compartments; Sam35 in mitochondria and Dpm1 in endoplasmic reticulum (ER). Sam35 is a component of SAM complex located in the mitochondrial outer membrane, while Dpm1 is a transmembrane protein of the ER membrane with its C-terminus exposed to the ER lumen (S4B Fig). We constructed the Sam35- and Dpm1-AlissAID strains by fusing GFP-tag at their C-termini. Immunoblots showed that Sam35-GFP was degraded, while Dpm1-GFP was not degraded in a 5-Ad-IAA-dependent manner (S4C Fig). Degradation of non-essential protein Sam35 caused no growth defects on 5-Ad-IAA-containing medium as did non-degradation of Dpm1 (S4D Fig). Taken together, these results indicate that antigen recognition sites must be exposed in cytosol or nucleus in the AlissAID system, as in the conventional AID system [1].

3. The size of mAID (7.4 kDa) tag is much smaller than GFP (26 kDA) and it will be more appropriate to compare the properties of ORF-FP vs ORF-mAID rather than use the awkward ORF-mAID-FP hybrid.

**Authors’ Responses**

We have attempted to generate an ssAID strain by adding the mAID-tag to the Neo1 protein in OsTIR1F74A-expressing cells. Despite much efforts, we were unable to obtain any correct clones. We hypothesized that basal degradation made it difficult to obtain mAID-tagged Neo1 in OsTIR1F74A expressing cells. Therefore, we used the mAID-GFP-tag instead of mAID tag to generate the ssAID clone.

4. Since the nanobody binding to FP protein is very tight, it is not clear why the basal degradation of ORF-mAID-FP hybrid will be higher as compared to ORF-FP coexpressed with AlissAID system.

**Authors’ Responses**

This comment is related to that of Reviewer #2. We provided a possible explanation about it in Discussion（Lines 247-249）

.

In AID systems, interactions occur in the cytosol. The cytosol has a variety of proteins, some of which might weaken the interaction between nanobodies and target proteins. Furthermore, the binding strength of nanobodies to the proteins depends on the nature of each nanobody.

＜Lines 247-249＞

It might be due to different mechanisms underlying interaction between OsTIR1^F74A^ and the target protein: AlissAID system provides an indirect interaction via mAID-Nb, while ssAID system provides a direct interaction.
